# Transcranial Focused Ultrasound Neuromodulation in Psychiatry: Main Characteristics, Current Evidence, and Future Directions

**DOI:** 10.3390/brainsci14111095

**Published:** 2024-10-30

**Authors:** Ahmadreza Keihani, Claudio Sanguineti, Omeed Chaichian, Chloe A. Huston, Caitlin Moore, Cynthia Cheng, Sabine A. Janssen, Francesco L. Donati, Ahmad Mayeli, Khaled Moussawi, Mary L. Phillips, Fabio Ferrarelli

**Affiliations:** 1 Department of Psychiatry, University of Pittsburgh, Pittsburgh, PA 15213, USA; keihania@upmc.edu (A.K.); sanguinetic@upmc.edu (C.S.); chaichiano2@upmc.edu (O.C.); hustonca@upmc.edu (C.A.H.); moorec26@upmc.edu (C.M.); chengc6@upmc.edu (C.C.); janssensa@upmc.edu (S.A.J.); mayelia@upmc.edu (A.M.); phillipsml@upmc.edu (M.L.P.); 2 Department of Health Sciences, University of Milan, 20142 Milan, Italy; 3 Department of Neurology, University of California, San Francisco, CA 94143, USA; khaled.moussawi@ucsf.edu

**Keywords:** non-invasive brain stimulation, transcranial focused ultrasound, psychiatric disorders

## Abstract

Non-invasive brain stimulation (NIBS) techniques are designed to precisely and selectively target specific brain regions, thus enabling focused modulation of neural activity. Among NIBS technologies, low-intensity transcranial focused ultrasound (tFUS) has emerged as a promising new modality. The application of tFUS can safely and non-invasively stimulate deep brain structures with millimetric precision, offering distinct advantages in terms of accessibility to non-cortical regions over other NIBS methods. However, to date, several tFUS aspects still need to be characterized; furthermore, there are only a handful of studies that have utilized tFUS in psychiatric populations. This narrative review provides an up-to-date overview of key aspects of this NIBS technique, including the main components of a tFUS system, the neuronavigational tools used to precisely target deep brain regions, the simulations utilized to optimize the stimulation parameters and delivery of tFUS, and the experimental protocols employed to evaluate the efficacy of tFUS in psychiatric disorders. The main findings from studies in psychiatric populations are presented and discussed, and future directions are highlighted.

## 1. Introduction

Noninvasive brain stimulation (NIBS) has traditionally been accomplished through electromagnetic techniques, including transcranial magnetic stimulation (TMS), transcranial direct current stimulation (tDCS), and transcranial alternating current stimulation (tACS) [1].

Recently, a new NIBS modality, known as transcranial focused ultrasound stimulation (tFUS), has emerged. The use of tFUS generates low-intensity acoustic waves, typically in the frequency range of 250 kHz to 700 kHz, to modulate neural activity [2]. These ultrasound waves can penetrate the skull non-invasively and can target both cortical and sub-cortical regions with millimetric precision (~1–5 mm) [3,4]. Furthermore, tFUS neuromodulation affects neuronal excitability through several mechanisms. Ultrasound waves induce mechanical strain gradients in neuronal membranes, altering the conformational state of membrane proteins and leading to depolarization. This depolarization facilitates action potential generation via thermodynamic membrane waves and activates mechanosensitive channels, thus promoting synaptic transmission. Additionally, the resonance of microtubules at ultrasonic frequencies can influence synaptic plasticity, while cavitation creates transient membrane pores that facilitate ion flow. Thermal modulation from ultrasound also activates thermosensitive ion channels, which can either promote or inhibit neuronal activity, depending on the tFUS parameters utilized [5,6]. Higher intensities and longer durations of stimulation tend to result in neuronal excitation, whereas lower intensities and shorter durations of stimulation are more likely to induce inhibitory effects [6,7,8]. However, stimulation protocols that consistently achieve excitatory or inhibitory outcomes are yet to be found and, therefore, this remains an area of active research [3,6,9,10]. 

This unique mechanism of action provides tFUS with several advantages over traditional electromagnetic NIBS techniques. For example, tFUS can reach deep neuronal structures (up to 8 cm) that are inaccessible to other non-invasive methods like TMS, which typically penetrates only 2–3 cm into the cortex with most coil types [1,5,10,11,12]. Unlike electromagnetic techniques, tFUS is unaffected by tissue conductivity, as ultrasound waves are mechanical [6,13]. Furthermore, because of the superior spatial resolution and depth of penetration, tFUS is better suited to study neural mechanisms in animal models than other NIBS techniques [8], thus allowing for more accurate targeting of different cell types, including excitatory neurons and inhibitory interneurons in both cortical and subcortical brain circuits [2,8]. In healthy individuals, several studies employing electrophysiological and neuroimaging assessments have recently demonstrated that tFUS can both enhance and inhibit neural activity in cortical as well as deep brain regions [6], with no severe adverse effects [6,14]. Mild symptoms were, however observed in less than 3.5% of subjects, including headache, mood deterioration, scalp heating, cognitive issues, neck pain, muscle twitches, anxiety, sleepiness, and pruritus [14,15]. Building on these promising initial findings, tFUS is being increasingly utilized to examine and modulate the functional properties of neural circuits in clinical populations, including psychiatric patients. 

In this narrative review, we will describe the key components of tFUS neuromodulation, including the tFUS setup and the neuronavigational tools used to precisely target deep brain regions (Section 2), as well as the sonication protocol parameters and simulations to optimize stimulation parameters (Section 3 and Section 4). We will also present sham strategies and methods for evaluating tFUS effects (Section 5 and Section 6). Next, we will review the current evidence on tFUS applications in psychiatric disorders, highlight the challenges of this technique, and discuss future directions (Section 7, Section 8 and Section 9).

## 2. tFUS Setup

**Figure 1:**
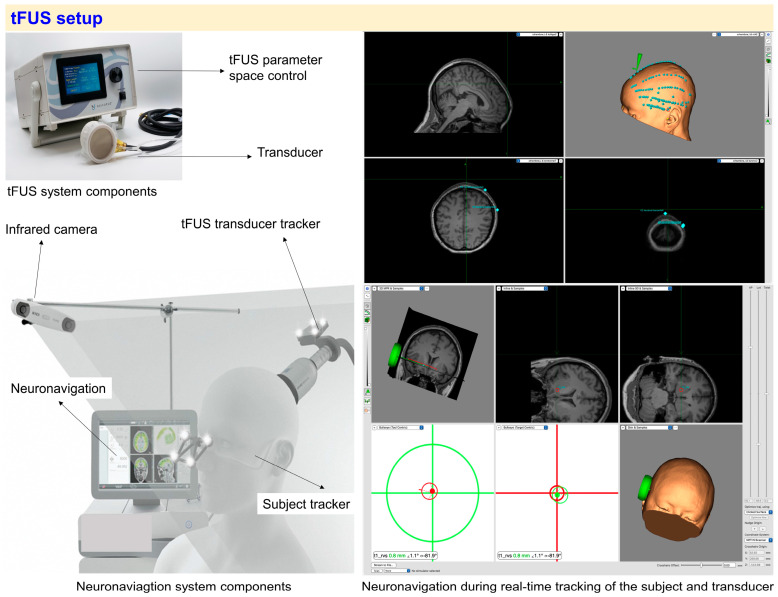
**Overview of key components of the tFUS setup.** (Left panel): The top left panel displays the main components of the tFUS setup, including the tFUS parameter space control unit and its transducer (image adapted from the BrainBox manufacturer, Cardiff, UK). The lower left panel shows the neuronavigation system, featuring infrared cameras for subject tracking and transducer positioning to assist in targeting during stimulation (image adapted from the NEUROLITH-TPS manufacturer, Tägerwilen, Switzerland). (Right panel): An example of real-time neuronavigation while tracking the subject and transducer with an infrared camera (images created using Brainsight software V2.5.3, Montréal, Canada). The top right panel shows scalp points (in green) that are taken for coregistering the brain MRI with the subject’s head position in real time. The bottom right panel displays the target and transducer positions, which are utilized to ensure an accurate and optimized stimulation.

The tFUS setup typically consists of two main subsystems: the tFUS system responsible for generating and delivering the ultrasound waves, and the neuronavigation system, which ensures accurate targeting and real-time monitoring of the stimulation (Figure 1) [1,2,3,10,16,17].

### 2.1. tFUS System Components

*tFUS control unit:* This unit is responsible for adjusting and controlling the stimulation parameters, including intensity, frequency, and duration of the ultrasound pulses (see Stimulation Parameters section) [10]. These parameters can be set manually, or via an external computer script or GUI connected to the control unit. 

*Transducer:* The transducer is the device that discharges the focused ultrasound waves. It converts electrical signals from the control unit into mechanical energy. Transducers come in various shapes, such as flat, concave, or phased-array designs, each allowing for different focal depths and spatial precision in targeting specific brain regions [18].

### 2.2. Neuronavigation System Components

*Infrared camera:* The infrared camera tracks the position of both the subject’s head and the tFUS transducer in real time. This is important because small movements by the subject during the procedure can lead to a misalignment of the ultrasound beam with the targeted brain region. By tracking both the subject and the transducer, the system ensures that the tFUS is delivered precisely to the desired brain regions throughout the procedure. The camera communicates with the neuronavigation system, which updates the position of the transducer relative to the subject’s brain in real time (Figure 1, right panel).

*tFUS transducer tracker:* This component tracks the exact position of the transducer during stimulation. The information is transmitted to the neuronavigation system to guarantee the alignment between the transducer beam trajectory and the targeted brain region (Figure 1, right panel). 

*Subject tracker:* The subject’s head and brain are tracked in real time using infrared markers. This ensures that any movement of the subject during the procedure is compensated for, maintaining the accuracy of the stimulation (Figure 1).

*Neuronavigation:* The neuronavigation system integrates all tracking data and provides a 3D visualization of the brain, helping to guide the tFUS transducer to the correct location (Figure 1).

## 3. tFUS Transducer and Stimulation Parameters 

**Figure 2:**
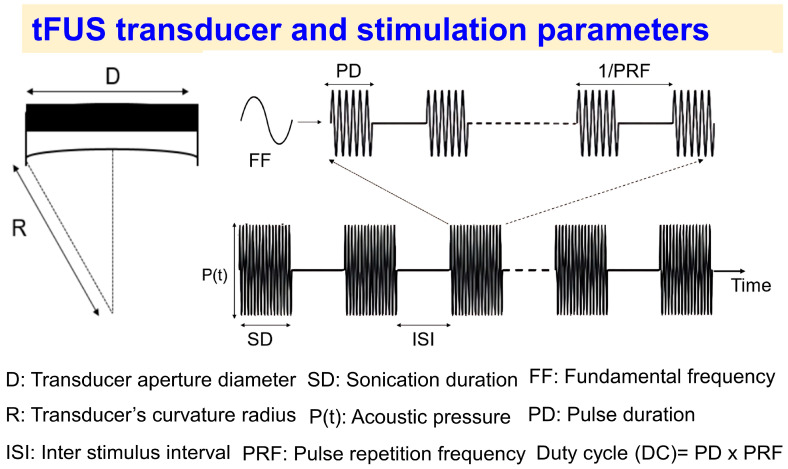
**Illustration of the geometry and parameters of the tFUS transducer.** Aperture diameter (D), curvature radius (R), fundamental frequency (FF), sonication duration (SD), acoustic pressure (P(t)), pulse duration (PD), interstimulus interval (ISI), and pulse repetition frequency (PRF) are displayed. These parameters define the sonication protocol.

The tFUS transducer and stimulation parameters are depicted in Figure 2. These parameters define the temporal and spatial characteristics of the acoustic pressure waveform, and the energy delivered at the targeted location.

Transducer parameters:

*Aperture diameter (D):* The diameter of the transducer’s active surface. A larger aperture generally leads to a narrower focal spot, which can improve the precision of the energy delivery. However, larger apertures also require more complex procedures to achieve accurate focal stimulation, making the process more technically demanding.

*Curvature radius (R):* The radius of the curvature of the transducer’s surface. A smaller curvature radius results in a more focused beam, but a very small curvature radius can reduce the transducer’s efficacy (e.g., decreased focal depth and focal volume). 

Combined, these transducer parameters determine the focality of the area that is targeted, as well as the intensity of the ultrasound beam [19]. 

The five main stimulation parameters that define a sonication protocol are as follows (Figure 2, upper right panel):

*1.*  *Fundamental frequency (FF, (kHz)):* This is the frequency of the ultrasound waves. Lower-frequency ultrasound waves generally penetrate deeper into tissues but provide a lower spatial resolution. Higher-frequency ultrasound waves have less penetration depth (i.e., the depth at which the ultrasound can stimulate neural tissue effectively) but offer higher spatial resolution (i.e., how accurately the ultrasound can target a specific region of the brain without affecting surrounding structures) and are more readily absorbed by tissues. The optimal FF for a tFUS study depends on the depth of the targeted tissue. For deep brain targets, higher FFs may be necessary to achieve sufficient penetration. However, the risk of excessive overheating must be carefully considered. Fundamental frequencies ranging from 200 to 650 kHz have been used in most human and animal studies [3].
*2.*  *Sonication duration (SD, (S)):* The duration of the ultrasound pulse train. Longer sonication durations can increase the energy delivered to the target, but they can also lead to tissue overheating and cavitation (i.e., the formation of microbubbles that can cause tissue damage).
*3.*  *Pulse repetition frequency (PRF, (Hz))*: The rate at which tFUS pulses are delivered within a train of pulses.
*4.*  *Duty cycle (DC, (%)):* This refers to the percentage of time the ultrasound is active during each pulse. In some studies, pulse duration (PD), i.e., the length of time for a single tFUS pulse, is reported instead of the DC. The DC can be calculated as the product of the PD and the PRF.
*5.*  *Intensity (I, (W/cm^2^)):* The amount of acoustic energy delivered per unit area, which is proportional to the square of the pressure (P(t)) and inversely related to medium density and sound speed (I∝P(t)^2^/(ρc), where P(t) is the pressure, ρ is the medium’s density, and c is the speed of sound in the medium). Since sound speed and density vary across tissue types, they affect intensity. For example, the sound speed is different in the scalp (~1450 m/s), the skull (~4000 m/s), and the brain (~1550 m/s). These differences impact how acoustic energy is absorbed and distributed across each tissue, thus influencing the depth and intensity of the energy delivered during tFUS applications [20].

Notably, tFUS protocols typically involve multiple pulse trains across sessions, each with a specific inter-stimulus interval (ISI) (Figure 2). To assess the safety of these protocols, the following parameters are commonly utilized:

*Spatial-peak temporal average (Ispta):* This is the average intensity at a specific point in space (i.e., spatial peak, where the ultrasound beam is most focused) over the entire pulse train duration, including the off periods of the pulsed ultrasound.

*Spatial-peak pulse average (Isppa):* This is the average intensity at the spatial peak during the active pulse (i.e., when the ultrasound is on), but does not include the intervals between pulses.

*Mechanical index (MI):* This estimates the likelihood of inertial (transient) cavitation, and it is measured as the ratio between the negative peak pressure and the square root of the fundamental frequency (i.e., higher frequencies are less likely to induce cavitation).

*Thermal index (TI):* This estimates the potential for tissue overheating during ultrasound exposure, as reflected by the ratio of the applied acoustic power to the power required to increase the tissue temperature by 1 °C.

These safety parameters are intensity-dependent, and the tFUS intensity can be measured in one of three ways:

*1.*  *Free-field intensity:* This is assessed in an open environment (e.g., water tank) before tissue interaction, and represents the transducer’s raw output.
*2.*  *Derated intensity:* This is adjusted for attenuation through the soft tissue, and it is calculated as $I_{\mathrm{derated}}=I_{\mathrm{free-field}}\times 10^{-\alpha \times d}$, where α is the tissue attenuation coefficient, and d is the tissue depth. The attenuation is typically based on a standard rate (i.e., the pressure measured in water derated by 0.3 dB/cm/MHz), which represents a general estimate of the amount of ultrasound energy that is absorbed or scattered by soft tissues. Of note, the derated intensity, as defined by the Food and Drug Administration (FDA) safety guidelines for diagnostic ultrasound [21], does not consider bone absorption.
*3.*  *In situ intensity*: this refers to the tFUS intensity at the depth of the brain target, accounting for factors like bone absorption and soft tissue interaction, which affect the ultrasound beam. This measurement reflects the actual intensity reached at the target.

The FDA safety guidelines for diagnostic ultrasound [21] aim to minimize the risks of cavitation and overheating, with the maximum permissible values of Ispta derated = 0.720 mW/cm^2^, Isppa free field = 190 W/cm^2^, and MI = 1.9 respectively. The International Transcranial Ultrasonic Stimulation Safety and Standards (ITRUSST) consensus on biophysical safety for transcranial ultrasonic stimulation reports that, for thermal effects, safety is ensured if any of the following criteria are met: the temperature increases by less than 2 °C, or the thermal dose is below 0.25 cumulative equivalent minutes at 43 °C (CEM43) [22].

## 4. Acoustic Simulations for tFUS Targeting

When conducting tFUS experiments on humans, both acoustic and thermal simulations should be performed. These simulations are important to ensure safety, but also to optimize tFUS targeting. Optimized acoustic and thermal simulations make it possible to account for the impact of the skull on the ultrasound beam’s shape and trajectory on the brain target and to ensure that the stimulation parameters comply with safety standards. 

**Figure 3:**
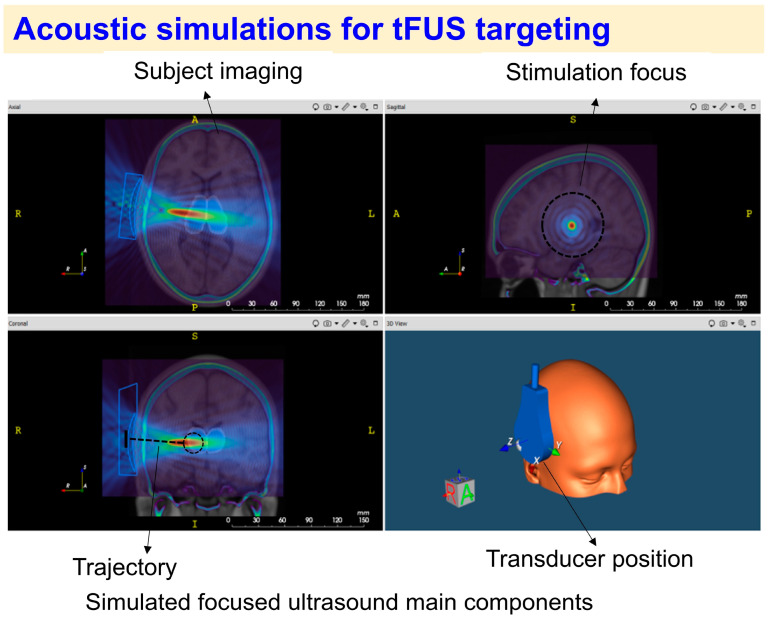
**Depiction of the acoustic simulation estimated for tFUS targeting of a brain region.** The simulation, which relies on individual neuroimaging data, determines the trajectory and target depth based on MNI or native space coordinates. The transducer position, acoustic focus, and predicted sonication protocol are simulated to assess both acoustic and thermal effects on the targeted brain region (image created with k-plan software (https://brainbox-neuro.com/products/k-plan) from BrainBox).

Acoustic simulation typically begins by generating a 3D model of the subject’s skull and brain using specialized software like SimNIBS [23], which segments the skull, skin, and brain. High-resolution T1- or T2-weighted MRI scans, along with CT scans (and/or zero-time echo (ZTE) [24] or pointwise encoding time reduction with radial acquisition (PETRA) scans [25]) can be used to improve skull reconstruction and simulation. The 3D model is then integrated with a neuronavigation platform to identify the brain target and the ideal beam trajectory (Figure 3). The ultrasound beam is simulated using tools that convert CT and MRI data into acoustic properties [18]. Computational models [18] embedded in software, such as k-Wave [26] or BabelBrain [27], ensure an accurate beam focusing on the targeted brain regions. Based on the subject’s anatomy, transducer configurations are optimized for ideal delivery. The optimization process involves iterative adjustments of the beam trajectory and transducer position to maintain the ideal path to the target. Thermal simulations are also conducted to ensure that temperature increases and mechanical index values remain within the safety limits established by the FDA for human tFUS procedures [21]. The description and discussion of the optimization procedures are also reported in [18]. However, despite the good acoustic and thermal simulation procedures currently available, the development and application of these simulations in tFUS procedures remain an active area of research. 

During tFUS delivery, a mechanical arm with a strapping device can be used to reach and stabilize the transducer on the optimal scalp position according to the acoustic simulation results. Furthermore, a frameless stereotactic localization system should be utilized to ensure that the targeted brain region is properly stimulated throughout the tFUS session. Several factors, including the software and computational modeling approach used, the acoustic simulations performed (e.g., the resolution of the acoustic simulations measured as points per wavelength), the placement of the transducer, and the location of the target should be considered to ensure consistency across sessions and tFUS studies. These factors should be reported in any tFUS study and included as covariates in analyses assessing the effects of tFUS delivery on the targeted brain region.

## 5. Different Sham Protocols Used in tFUS Studies

**Figure 4:**
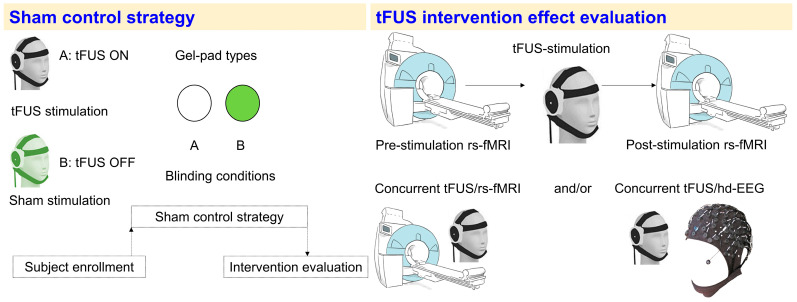
**Sham strategies and evaluation methods used in tFUS studies.** (Left panel): The illustration presents the subject enrollment and blinding process, in which participants are randomized into either active (tFUS ON) or sham (tFUS OFF) conditions. The choice of gel-pad type can further enhance blinding by minimizing differences in scalp perception between active and sham sessions (the 3D visualization of the transducer on the head model was adapted from [62]). (Right panel): Different experimental approaches for evaluating the effects of tFUS on brain function. These include offline methods, such as pre- and post-tFUS resting-state fMRI (rs-fMRI) or hd-EEG (EEG electrode cap image adapted from Compumedics Neuroscan company, Victoria, Australia), and online methods using concurrent tFUS with either rs-fMRI or hd-EEG to capture immediate neurophysiological changes induced by the stimulation.

As for other NIBS modalities, developing an adequate sham is important for both research and clinical studies involving tFUS [28]. Since tFUS is a relatively novel technology, an optimal sham condition is yet to be fully established. A well-designed tFUS study should address three key issues to ensure that the active tFUS and sham conditions are indistinguishable (Figure 4, left panel):

1. Auditory perception of the pulsed ultrasound: in tFUS studies, participants can report hearing a sound time-locked to the active stimulation, likely due to bone-conducted flexural waves reaching the cochlea during tFUS [29,30].
2. Tactile sensation: in tFUS studies, participants also experienced tactile sensations at the position in the scalp where the transducer was placed [29,31].
3. Thermal sensation: typically overheating, which is usually reported on the scalp region under the transducer during active tFUS [32,33].

Among those, the most commonly reported experience is auditory perception, albeit not in all tFUS studies (e.g., [34]). The sham conditions that have been employed in tFUS studies so far are the following:

1. Positioning the transducer over the participant’s head without delivering the ultrasound stimulation [30,35,36,37].
2. Turning the transducer upside down so that stimulating side point away from the participant’s scalp [38,39,40,41].
3. Using a cap and/or a high-impedance disk/gel-pad between the transducer and the scalp of the participant to block the ultrasound wave [31,42,43].
4. Increasing the distance between the transducer and the scalp (i.e., detaching the transducer) [42].
5. Defocusing the acoustic waves [44,45].
6. Using an active control (i.e., stimulating a brain region not involved with the tFUS primary target region [46],) or a volume not containing neuronal cells (i.e., stimulating the cerebral ventricles) [47].

Each sham protocol has its advantages and its limitations. For example, blocking the ultrasound with a gel pad or detaching the transducer may successfully eliminate stimulation but fails to mimic the auditory or thermal sensations of active tFUS. On the other hand, stimulating an active control region may lead to patterns of activations that partially overlap with the tFUS of the targeted brain region. The most significant challenge remains to achieve indistinguishable sensory experiences between the sham and active conditions that help preserve the blinding [48]. It is also necessary to develop standardized sham conditions that can be applied across different protocols and research sites, thus ensuring reliability and reproducibility across tFUS studies.

## 6. Evaluating the Effects of tFUS 

The effects of tFUS on brain activity can be evaluated utilizing various neuroimaging and electrophysiological techniques, both during and after stimulation in healthy individuals (Figure 4, right panel) [6,9,34,42,49,50,51,52,53]. Several neuroimaging studies have used resting-state functional MRI (rs-fMRI) to examine changes in brain connectivity following tFUS stimulation. These fMRI studies showed that tFUS can modulate neuronal activity in various brain networks by either increasing or decreasing their connectivity. For example, tFUS targeting the right prefrontal cortex led to reduced resting-state functional connectivity in networks associated with emotional regulation [34]. In another study [29], tFUS applied to the somatosensory cortex or the thalamus enhanced fMRI connectivity within sensorimotor and sensory integration networks. Task-based fMRI has also been utilized to investigate the effects of tFUS on neuronal activity. For instance, targeting the motor cortex increased local BOLD signal activity during a cued finger-tapping task [54], while tFUS applied to the visual cortex activated both the sonicated area and broader visual processing networks during visual tasks [55]. 

EEG studies have shown that tFUS resulted in changes in evoked potentials and spectral power through several mechanisms, including mechanical perturbation, neuronal pathway influence, mechanosensitive ion channel effects, and/or selective frequency band modulation [51,56]. For example, tFUS of the right inferior frontal gyrus of the lateral prefrontal cortex modulated mid-frontal EEG theta power by altering its relationship with self-reported effort and worrying [57], while another study reported increased EEG beta power and decreased EEG theta activity on the bilateral medial prefrontal cortex during and after tFUS relative to sham conditions [58]. There is currently a lack of evidence from high-density electroencephalography (hd-EEG) studies on tFUS. Future research could benefit from analyzing the time-frequency information of neural activity following tFUS using source localization techniques. Additionally, acquiring hd-EEG data after tFUS administration would provide more detailed insights into the neurophysiological effects of this neuromodulation technique [59]. 

Concerning the effects of tFUS on neural activity during the stimulation, concurrent tFUS-fMRI scans of the primary visual cortex have shown increased activation in both the sonicated area and related visual processing networks [55], whereas decreased BOLD signals in the targeted left globus pallidus and large-scale cortical networks were reported by another simultaneous tFUS/fMRI study [60]. Concurrent tFUS-EEG recordings of the primary somatosensory cortex reported a significantly attenuated amplitude of somatosensory evoked potentials elicited by median nerve stimulation along with a change in the spectral power of sensory-evoked EEG oscillations [49]; furthermore, a computational model applied to simultaneous tFUS-EEG data on the somatosensory cortex showed local changes in phase rate of beta and gamma frequencies and in phase distributions within the beta band of early sensory-evoked activity [51]. 

In addition, magnetic resonance acoustic radiation force imaging (MR-ARFI) is an innovative technique used to visualize and confirm the targeting of tFUS in large animal models [61]. MR-ARFI uses motion encoding gradients applied simultaneously with the tFUS pulse to image the microscopic displacements generated by ultrasound sonication in the brain tissue. 

Combined, these findings indicate that tFUS can enhance or inhibit local target function, as shown by task-based fMRI, and effectively engage different brain networks in both task-based and resting-state fMRI. Time-frequency changes in brain function can be observed through neurophysiological recordings, such as EEG, and more innovative techniques like MR-ARFI specifically visualize and confirm the physical impact of tFUS on brain tissue. Altogether, these readouts demonstrate the ability and efficacy of tFUS in target engagement in healthy individuals. In the next section, we will review studies employing tFUS in psychiatric populations.

## 7. Overview of Psychiatric Studies Using tFUS

An increasing number of studies have been employing tFUS in individuals with psychiatric disorders (see clinicaltrials.gov and [9]), although the published work is relatively limited (Table 1). This work includes six studies on depression, one on schizophrenia, one on generalized anxiety disorder, one on substance use disorder, and one on autism spectrum disorder.

### 7.1. Depression

Depression has been, so far, the most studied psychiatric disorder for tFUS application, with three double-blind, sham-controlled studies [63,64,65], a tFUS vs. waiting list study [66], and two case reports [44,45]. 

Reznik et al. [64] conducted a randomized, double-blind, sham-controlled, parallel arm pilot trial to evaluate the effect of repetitive sessions of tFUS in young adults (N = 24 participants) with mild-to-moderate depressive symptoms. Each participant underwent an active or sham (N = 12 vs. N = 12) 30 s tFUS session (FF 0.5 MHz, pulse duration 65 µs, PRF 40 Hz, duty cycle 0.26%) over the right fronto-temporal cortical area for five consecutive days. No significant effects were observed on mood in the tFUS vs. sham group, although a trend towards a reduction in worry feelings was observed following the tFUS condition. 

Riis et al. [63] targeted the bilateral subcallosal cingulate cortex (SCC) in a double-blind, sham-controlled, crossover trial. Individuals (N = 22) with either major depressive disorder (MDD) or bipolar disorder (BD) experiencing a long depressive episode were enrolled. A protocol hypothesized to have inhibitory effects was used: 5 ms pulse duration, 50% DC, PRF 100 Hz, 30 ms on and 1.4 s off, for 60 s, repeated for a cumulative duration of around 1 h, including one active and one sham session. The clinical results were evaluated and compared between the groups (N = 10 active, N = 12 sham) after the first stimulation session: immediately after the session, at 24 h, and at 7 days. At 24 h, the reduction in the 6-item Hamilton Depression Rating Scale (HDRS-6) scores was greater in the active group compared to the sham, and this reduction was present in a subset of participants even at the 7-day follow-up; the fMRI analysis revealed that SCC activation was reduced after active tFUS at a group level (N = 16), while the sham stimulation (N = 8) did not affect SCC activity. Whole-brain analysis confirmed that only the SCC was significantly inhibited by active tFUS, while the left ventrolateral prefrontal cortex (VLPFC) and bilateral temporal cortex were activated. 

Oh and colleagues [65] assessed the efficacy of repeated sessions of a tFUS protocol (tone burst duration of 1 ms, duty cycle of 50%, sonication duration 300 ms) on the left dorso-lateral prefrontal cortex (DLPFC) of patients (N = 23 participants) with major depressive disorder (MDD) in a double-blind, randomized, sham-controlled, parallel-arm trial, with N = 11 in the active arm and N = 12 in the sham arm. The authors found a significant reduction in Montgomery–Åsberg Depression Rating Scale (MADRS) scores, but not in self-rated depression scales, in the tFUS vs. sham groups. Furthermore, no changes in the functional connectivity (FC) of the DLPFC (i.e., the sonicated area) or the subgenual anterior cingulate cortex (sgACC) were observed in either group, while an increase in FC was reported between different sgACC sections and several brain regions after the tFUS, but not the sham condition. 

Cheung et al. (2023) [66] evaluated the efficacy of transcranial pulse stimulation (TPS), a form of tFUS that delivers single ultrashort (3 µs) ultrasound pulses with 0.2–0.25 mJ/mm^2^ energy levels and 4–5 Hz pulse repetition frequency, over the left DLPFC vs. a waiting list control group in MDD patients with a single-blind, randomized, pilot study (N = 15 participants TPS, N = 15 participants waiting list). The authors reported a significant reduction in Hamilton Depression Rating Scale (HDRS) scores in the TPS group, although a significant placebo effect could not be ruled out due to the absence of a sham control group [66].

In a case report preceding the abovementioned clinical trial [63], the same team [45] evaluated the effects of a long session (i.e., 64 min) of tFUS for the anterior and posterior subcallosal cingulate cortex (SCC), as well as for the pregenual cingulate. The FF was 650 kHz, the waveform was continuous with 30-millisecond ON periods followed by 4second off periods, with an average duration of 2 min, repeated around 10 times for each target. The patient, a 30-year-old woman with severe, treatment-resistant, non-psychotic depression showed an improvement in depressive symptoms within 24 h of sonication, which lasted for several weeks after the treatment. Concurrent tFUS/fMRI also showed decreased BOLD signals at the sonicated targets during tFUS, but not during the unfocused sham condition.

In Fan et al.’s study [44], a man with treatment-resistant depression underwent repeated sonication of three targets: The ventral capsule (VC), the bed nucleus of the stria terminalis (BNST), and the anterior nucleus of the thalamus (ANT) bilaterally. The FF was 500 kHz, and the stimulation parameters were as follows: 25 Hz PRF, 13% duty cycle, and 300 s pulse train duration, with an intensity (42–50 W/cm^2^ Isppa at the target) considerably higher than those employed in other tFUS studies. Only the tFUS of the ANT significantly reduced subjectively reported depressive symptoms when compared to unfocused ultrasound stimulation. Furthermore, compared to the unfocused stimulation, active tFUS resulted in a FC reduction within the default mode network, which was hyperactive in the study participant at baseline. 

### 7.2. Schizophrenia

A recent double-blind, randomized, sham-controlled trial investigated the efficacy of 15 daily sessions (i.e., each workday for 3 weeks) of tFUS over the DLPFC in N = 26 (N = 13 in the active group and N = 13 in the sham group) patients with chronic schizophrenia and marked negative symptoms [67]. The stimulation protocol, which was previously found to have excitatory effects on the motor cortex [68], included the following: PRF 100 Hz, duty cycle 5%, and 0.5 s sonication duration with 8 s of interstimulus interval. In the active tFUS, the SCZ patients had a significant reduction in the Scale for the Assessment of Negative Symptoms (SANS) score at the end of the treatment vs. baseline [67], while no changes were found in the sham group. An improvement in the continuous performance task (CPT) performance, but not in other tasks, was also observed only after active tFUS. 

### 7.3. Anxiety

Preliminary results from an open-label pilot study showed a significant reduction in anxiety symptoms, assessed with the Hamilton Anxiety Rating Scale (HAM-A) and Beck Anxiety Inventory (BAI), in N = 25 participants with treatment-resistant generalized anxiety disorder (trGAD) after 8 weekly tFUS sessions (FF 650 kHz, pulse width 5 ms, duty cycle of 5%, PRF 10 Hz, 30s of stimulation followed by 30s ISI, for 10 min) over the right amygdala [69]. Although promising, these findings should be considered cautiously given the open-label design and the potentially high placebo response in participants with anxiety disorders [28]. Moreover, several participants had comorbid MDD or obsessive-compulsive disorder (OCD) [70]. 

### 7.4. Substance Use Disorder

An open-label pilot trial (N = 4 participants) employing tFUS (pulse duration (on/off) 100/900 ms, duty cycle 3.3%, repetition times (on/off) 5/10 s) over the bilateral nucleus accumbens (NA) showed a reduction in cue-induced and daily substance craving both immediately and at long-term follow-up in patients with substance use disorder (SUD) [71]. This trial also found that intensities considerably higher (60 and 90 watt of “maximal instantaneous output power”) than those employed in previous human studies appeared to be safe and tolerable, with adverse events (head pain (N = 2), headache (N = 1), nausea (N = 1), and scalp swelling (N = 1)) of mild intensity that remitted on the same day. 

### 7.5. Autism Spectrum Disorder

A double-blind, randomized, sham-controlled study applied active TPS (N = 16) or sham TPS (N = 16) on the right temporo-parietal junction in adolescents with autism spectrum disorders (ASD) every two days for two consecutive weeks [72]. In the active, but not sham TPS, significant improvements in several ASD symptom domains, including emotional response, relating with other people, adaptation to change, fear of nervousness, and verbal communication were observed, and these improvements were sustained for up to 3 months. It is, however, important to point out that, while all the participants in the TPS group believed that they had the active treatment, only 43.75% of the participants in the sham group thought that they had received TPS.

**Table 1:** Overview of currently published tFUS studies in psychiatric patients.

Authors, Year	Experimental Design & Psychiatric Group	Sample Size (N Female)	Sham Condition	Target Location	tFUS System and Software	Stimulation Parameters and tFUS Protocol	Assessments of tFUS Effects (rs-fMRI, etc.)	Type of Effect (Excitatory, Inhibitory)	Main Findings	Tolerability/Side Effects
Reznik et al., 2020 [64]	Randomized, double-blind, two-armed, sham-controlled. Mild-to-moderate depression.	24 (16) 12 actives 12 shams	Transducer in the same position but no ultrasound emitted	Right fronto-temporal cortex	Neurotrek U+™, (Neurotrek Inc., Los Gatos, CA, USA). Acoustic simulations: k-Wave.	Estimates for the TI (0.6), MI (0.9), peak negative pressure (MPa = 0.65), Ispta = 71 mW/cm^2^, Isppa = 14 W/cm^2^. 30 s session. Five laboratory visits over seven days of Active TUS or Sham.	Clinical scales. 3 times per visit: VAMS At the end of each session: BDI-II OASIS First and last day of study, post-tFUS: RRS PSWQ	Inhibitory	Lack of different effects on mood for active tFUS vs. sham. A trend-level reduction in worry feelings after the active tFUS condition. At one month follow-up after the end of tFUS sessions, no between-group differences in depressive or anxiety symptoms (BDI, OASIS).	Was safe. No other details reported.
Oh et al., 2024 [65]	Randomized, double-blind, two-armed, sham-controlled. Major depressive disorder (MDD).	23 (13) 11 (6) active 12 (7) sham	Transducer in the same position but no ultrasound emitted	Left dorso-lateral prefrontal cortex (lDLPFC)	NS-US100; (Neurosona Co. Ltd., Seoul, Republic of Korea) operating at 250-kHz FF. Acoustic stimulations: k-Wave.	Tone burst duration: 1 ms; duty cycle: 50%; sonication duration: 300 ms. Estimated in situ Pr 135 kPa (corresponding mechanical index: 0.27), in situ acoustic intensity: 600 mW/cm^2^ average spatial-peak pulse intensity (corresponding spatial-peak temporal average intensity of 300 mW/cm^2^). 20 min/session, three times a week over two weeks.	Clinical and neuro-psychological scales. Primary outcome: changes in MADRS scores across sessions. Others: CANTAB QIDS SR STAI Assessments at baseline, 1 day and 2 weeks after the end of the tFUS sessions. fMRI: 5 min resting state fMRI at baseline and 2 weeks after the end of the tFUS sessions.	Excitatory	Reduction in MADRS scores more profound in the verum group, immediately after the completion of the series of tFUS sessions and maintained for at least two weeks. STAI scores also had greater decreases in the verum group. No changes in FUS-mediated FC in the stimulated left DLPFC area (within and between groups). Increase in FC between different sgACC portions and several brain regions after active tFUS, but not sham. No correlation between the changes in MADRS scores and FC strengths.	TFUS was well tolerated, without adverse events and undesirable side effects. No tFUS-related sound perceived.
Riis et al., 2024 [63]	Double-blind, randomized, sham-controlled, cross-over. Major depressive disorder (N = 20) or bipolar disorder (N = 2). Current moderate-to-severe depressive episode without psychotic features, lasting at least 2 months.	22 (14). 10 (6) active tFUS first. 12 (8) sham first	Stimulation with the same waveform and pressure amplitude but unfocused	Bilateral cingulate (SCC)	Two spherically focused 126-element arrays, operating at 650 kHz FF, driven by Vantage256 (Verasonics) power unit.	During rs-fMRI (target engagement): Estimated peak pressure at target 1 MPa (31.1 W/cm^2^ following skull correction), 5 ms pulse duration, 50% DC, PRF 100 Hz, 30 ms on and 1.4 s off, for 60 s. 10 min with alternation of 1 min on and 1 min off. Out of MRI (treatment protocol): Same protocol but session lasting, cumulatively, around 1 h, over three SCC targets	Clinical scales: Primary outcome: compared to baseline, PANAS-X Sadness immediately after first stimulation, HDRS-6 score 24 h and 7 days after first stimulation. FMRI: BOLD signals during sonication.	Inhibitory	Clinical: Significantly higher reduction in HDRS-6 in active vs. sham group at 24 h. No other significant differences between groups. fMRI (N = 16, target engagement): After active tFUS, significant decrease in target activity at group-level analysis and in a subgroup (N = 5) at individual level.	No Serious Adverse Events (SAEs), no switch to mania or hypomania. In the period 24–72 h following real SCC stimulation, in 2 participants, worsening of depression and suicidal ideation, resolved within 2 weeks. Most common side effects (at follow-up visit 24 h after stimulation): depressed mood (active, 62%; sham 67%), headache (active, 57%; sham, 67%), anxiety (active, 57%; sham 52%). Suicidal thoughts (active 29%; 24% sham).
Cheung et al., 2023 [66]	Single-blind, randomized, sham-controlled. Major depressive disorder (MDD).	30 (22) 15 (11) intervention 15 (11) WC	Waitlist control (WC)	Left dorso-lateral prefrontal cortex (lDLPFC)	Transcranial Pulse Stimulation (TPS^®^ system, NEUROLITH, Storz Medical, Tägerwilen, Switzerland).	Single ultrashort (3 µs) ultrasound shockwave pulses, 300 pulses in each session (total: 1800 pulses). 0.2–0.25 energy levels (mJ/mm^2^) and 2.0–4 Hz pulse repetition frequencies. Both for the TPS group and the WC group: 6 sessions of 30 min with 3 sessions per week on alternate days, for 2 consecutive weeks.	Clinical, functional, and neuro-psychological scales: Primary outcome: HDRS-17. Secondary outcomes: Chinese version of SHAPS IADL. Chinese version of MoCA forward and backward digit span. Trail making test A and B. Both groups measured at baseline (T1), immediately after the intervention (T2), and at the 3-month follow-up (T3).	N.R.	Immediate post-stimulation significant reduction in depressive symptoms with large effect size. The effect of TPS on primary and secondary outcomes was maintained at the 3-month follow-up assessment. TPS improved participants’ cognition when compared with baseline.	No serious adverse events. Headache (4%), pain or pressure (1%), and mood deterioration (3%) reported. These symptoms lasted up to 2 h after the stimulation.
Riis et al., 2023 [45]	Case report. Treatment-resistant depression.	1 (1)	Stimulation with same waveform and pressure amplitude but unfocused	Posterior cingulate, anterior cingulate, pregenual cingulate	Diadem.	For each target: FF 650 kHz continuous wave, 30-millisecond on 4-s off periods, 0.8% duty cycle; average duration of a stimulation epoch was 2 min (range 20–180 s). Each target was sonicated 10 times with randomized order between sites, 30 stimulation epochs, for a total duration of 64 min. Estimated peak pressure at target was 1.0 MPa.	Clinical scales: HDRS-6 scores up to 44 days following the stimulation. FMRI BOLD signals: during the stimulation.	Inhibitory	Pre-sonication HDRS-6 score of 11 fell to 0 the day following the stimulation. The patient remained in remission (HDRS-6 = 0) for at least 44 days, the last assessed timepoint. Five months after the stimulation, patient experienced a recurrence of depressive symptoms. Significant decrease in fMRI BOLD activity at the target, only observed during active stimulation.	No adverse events.
Fan et al., 2024 [44]	Case report. Treatment-resistant depression.	1 (0)	Unfocused stimulation control	Ventral capsule (VC), bed nucleus of stria terminalis (BNST), anterior nucleus of the thalamus (ANT).	ATTN201 wearable device, equipped with dynamic steering (128 transducer elements; Attune Neurosciences, Inc., San Francisco, CA, USA). Acoustic and thermal simulations: k-Wave.	FF 500 kHz, PRF 25 Hz, duty cycle 13%, pulse train duration 300s (5 min). Simulated Isspa ranged from 42.2 to 50.2 W/cm^2^ at the target for active conditions and <2 W/cm^2^ for unfocused control. In exploratory phase for target selection: stimulation alternating between left and right lateralized regions every 15 min with 10 min of no stimulation interval. Eight total stimulations, 4 on the left and 4 on the right side.	Clinical scales: VAS-D HAMD-6. Resting-state fMRI: baseline and following ANT and unfocused stimulation.	Inhibitory	Thalamic tFUS elicited subjective reduction in depressive symptoms. tFUS followed by a decrease in Default Mode Network (DMN) connectivity.	TFUS was well tolerated by the participant, no adverse events.
Zhai et al., 2023 [67]	Double-blind, randomized, two-armed, sham-controlled. Schizophrenia patients with predominantly negative symptoms.	32 (16) 16 (7) active 16 (9) sham 26 patients completed the RCT	Transducer in the same position but no ultrasound emitted	Left dorso-lateral prefrontal cortex (lDLPFC)	Immersion-type focused ultrasound transducer (V391-SU, Olympus NDT, Waltham, MA, USA). driven by a custom-made TUS system.	Focal length 3.8cm, FF 500 kHz, pulse duration 0.5 ms, PRF 100 Hz, DC 5%, 0.5 s on and 8s off, Isspa.0 (water) 8.086 W/cm^2^, Ispta.0 (water) 0.404 W/cm^2^. 15 sessions on workdays for three consecutive weeks.	Clinical and neuro-psychological scales: Primary outcome: SANS scores, pre- and post-. Secondary outcomes: PANSS C-BCT (TMT-A, Symbol Coding, CPT, Digital Span).	Excitatory	SANS scores decreased significantly after tFUS in the active group. No significant reduction observed in the sham group. Results from the PANSS total score portrayed a similar trend. Active tFUS was followed by an improved performance in CPT, but not in the TMT-A, Symbol Coding, and Digital Span tasks.	TFUS was generally well tolerated. No serious adverse events (SAEs) were reported. Dizziness during the procedure (N = 3, in the sham group), difficulty falling asleep (N = 2 in the active group) resolved within 7 days.
Mahdavi et al., 2023 [69]	Open-label, non-controlled. Moderate-to-severe treatment-resistant generalized anxiety disorder.	25 (11)	No sham	Right amygdala	Brainsonix Pulsar 1002.	FF 650 kHz, pulse duration 5 ms, duty cycle 5%, PRF10 Hz, I sppa.3 W/cm^2^, I spta.3 719.73 mW/cm214.39. mechanical index 0.75, peak negative pressure 0.61 MPa. 30s on and 30 s off for 10 min. Eight weekly tfUS sessions.	Clinical scales: Primary outcome: HAM-A BAI Assessed at baseline and after protocol completion.	Inhibitory	Post/pre-tfUS significant decrease in anxiety as measured by the HAM-A.	All patients tolerated the procedures, no notable side effects or adverse events.
Mahoney et al., 2023 [71]	Open-label, sham-controlled, cross-over. Substance use disorder.	4 (1)	Transducer in the same position but no ultrasound emitted	Left and right nucleus accumbens (NA)	ExAblate Neuro Type 2 (Insightec) device/system; the transducer helmet array comprised > 1000 ultrasound transducers.	Pulse duration (on/off) 100/900 ms, duty cycle (%) 3.3%, repetition times (on/off) 5/10 s, intensity (W/cm^2^): 55 or 80. Sham: 5 min to the left NAc, followed by 5 min to the right NAc. Sham tFUS delivered first in all participants. Active: two 5-min sessions to the left NAc, followed by two 5-min sessions on the right NAc	Clinical measures: Cue-induced substance craving (acute tFUS effects). Substance craving (ecological momentary assessment). Cue-induced substance craving (prolonged effects of tFUS sonication). Urine toxicology and self-reported alcohol and substance use.	N.R.	In the two participants receiving the enhanced tFUS dose (80 W), cue-induced craving for several substances decreased acutely and remained reduced for 90 days after receiving one tFUS sonication.	No alterations at structural follow-up MRIs. Mild-to-moderate side effects related to the procedure reported: head pain (*n* = 2), headache (*n* = 1), nausea (*n* = 1), scalp swelling (*n* = 1).
Cheung et al., 2023 [72]	Two-armed, double-blind, randomized, sham-controlled. Adolescents with autism spectrum disorder (ASD).	Active: 16 (3) Sham: 16 (2)	The sham procedure was identical to the active, except for the silicone oil being replaced by an air-filled standoff cushion in the handpiece	Right temporo-parietal junction	Transcranial pulse stimulation (TPS^®^ system, NEUROLITH, Storz Medical AG, Tägerwilen, Switzerland).	Single ultrashort (3 µs) ultrasound shockwave pulses. 0.2–0.25 energy levels (mJ/mm^2^) and 2.0–4 Hz pulse repetition frequencies. Each TPS session lasted 30 min. Each participant received 800 TPS pulses. 6 verum TPS or sham sessions, 3 sessions per week, for 2 consecutive weeks.	Clinical and neuro-psychological scales: Primary outcome: CARS. Secondary outcomes: AQ ASAS SRS TMT VFT Stroop test Digit Span Test (forward and backward) CGI. Assessed at baseline, immediately after intervention (2 weeks), and at 1- and 3-month follow-ups.	N.R.	TPS over temporoparietal junction was effective in reducing some core symptoms of ASD (i.e., relating with people, emotional response, adaptation to change, fear of nervousness, verbal communication).	Around 1/3 of participants (*n* = 5) in the active group reported mild to moderate headaches during the stimulation, which subsided immediately after the session. No side effects in the sham group.

Fundamental frequency (FF), thermal index (TI), mechanical index (MI), spatial-peak pulse-average intensity (Isppa), spatial-peak temporal-average intensity (Ispta), free-field spatial-peak pulse-average intensity (Isppa.0), free-field spatial-peak temporal-average intensity (Ispta.0), spatial-peak pulse-average intensity derated to account for the acoustic attenuation in soft tissues (Isppa.3), spatial-peak temporal-average intensity derated to account for the acoustic attenuation in soft tissues (Ispta.3), duty cycle (DC), pulse repetition frequency (PRF), interstimulus interval (ISI), transcranial pulse stimulation (TPS), resting-state functional magnetic resonance imaging (rs-fMRI), Montreal cognitive assessment (MoCA), Positive and Negative Symptoms Scale (PANSS), Trail Making Test (TMT), Verbal Fluency Test (VFT), Clinical Global Impression (CGI), Social Responsiveness Scale (SRS), Childhood Autism Rating Scale (CARS), Beck Anxiety Inventory (BAI), Hamilton Anxiety Inventory (HAM-A), Chinese Brief Cognitive Test (C-BCT), Trail making test, part A (TMT-A), continuous performance test (CPT), Visual Analog Scales of Depression (VAS-D), 6-item Hamilton Depression Rating Scale (HAMD-6 or HDRS-6), subcallosal cingulate cortex (SCC), Positive and Negative Affect Schedule (PANAS-X), instrumental activities of daily living (IADL), Chinese version of the Snaith–Hamilton pleasure scale (SHAPS), 17-item Hamilton rating scale for depression (HDRS-17), Visual Analogue Mood Scales (VAMS), Beck Depression Inventory-II (BDI-II), Overall Anxiety Severity and Impairment Scale (OASIS), Ruminative Responses Scale (RRS), Penn State Worry Questionnaire (PSWQ), Cambridge Neuropsychological Test Automated Battery (CANTAB), Montgomery–Åsberg Depression Rating Scale (MADRS), Quick Inventory of Depressive Symptomatology–Self Report (QIDS-SR), State-Trait Anxiety Inventory (STAI), Autism Spectrum Quotient (AQ), Australian Scale for Asperger’s Syndrome (ASAS).

## 8. Discussion

In this review, we described the main features of tFUS neuromodulation and explored the current state of this intervention in psychiatric disorders. The tFUS studies on depression targeted both cortical (i.e., left DLPFC, right frontotemporal cortex) and subcortical (i.e., SCC, and ANT) regions, a unique advantage of tFUS over traditional non-invasive brain stimulation techniques, including TMS or tDCS, which do not allow the direct targeting of deep brain structures in humans [8]. These studies showed some improvements in depressive symptoms, sometimes lasting weeks or months after stimulation, although one study [64] failed to report beneficial effects on mood. Furthermore, only four studies utilized fMRI alongside clinical assessments, with mixed findings on brain connectivity changes after active tFUS [44,45,65], while in anxiety and SUD patients, the targeted brain regions, the amygdala and nucleus accumbens, respectively, induced a reduction in clinical symptoms after active stimulation; however, no neural activity measures were examined in any of those studies. Further research is therefore needed to confirm these outcomes, particularly through neurobiological measures like changes in brain activity and connectivity.

More generally, although they are encouraging, these findings leave several unanswered questions, including how to optimize the stimulation parameters and delivery of tFUS, how to best account for possible confounds (i.e., placebo effects), and how to properly assess tFUS effectiveness and safety in psychiatric populations. In what follows, we provide some suggestions on how to begin answering these questions. 

### 8.1. Standardizing the Reports of tFUS Protocol Parameters, Including Safety Metrics, to Facilitate Aross-Study Comparisons and Optimize Sonication

The first important challenge pertaining to the discovery of the optimal stimulation parameters and delivery of tFUS is the inconsistency in reporting key parameters, including the in situ intensity, mechanical index, and thermal index across studies. The absence of such information negatively affects between-study comparisons and limits the reproducibility of findings. Standardizing the reports of tFUS parameters, as recommended by the ITRUSST consortium [19], is therefore an essential step toward facilitating across-study comparisons and optimizing sonication effects. While tFUS mechanisms are not fully understood, evidence suggests that the stimulation’s effects may depend on the specific parameters used, such as the duty cycle, which influences whether tFUS has excitatory or inhibitory effects [73]. Thus, there is a need to investigate the impact of variables using different computational models to estimate the effects of tFUS on the targeted brain regions, which, in turn, may lead to more effective modulation of these regions’ neural activity. 

The ITRUSST has also developed an expert consensus on biophysical safety considerations for tFUS recently [22]. This guideline, informed by the diagnostic ultrasound literature, aims to offer some indications, but these are not intended to replace regulatory standards. They propose safety thresholds, including maintaining the MI or transcranial mechanical index (MItc) below 1.9 and limiting thermal effects, ensuring that temperature rises remain below 2 °C or that thermal doses are under 0.25 CEM43. Of note, although several tFUS studies were conducted before the introduction of the ITRUSST recommended safety guidelines, and some of the studies reviewed here did not report critical parameters, such as in situ intensity values and mechanical indices, a key positive takeaway is the absence of severe adverse events and the overall good tolerability across all the studies in psychiatric patients, with only mild, transient side effects clearly related to active tFUS stimulation reported in a few cases. This finding aligns with results from studies on healthy participants [14,15], further reinforcing the safety profile of tFUS. Nonetheless, establishing standardized safety guidelines and consistent reporting protocols for studies involving non-clinical and clinical populations is crucial to advancing tFUS research and its clinical applications.

### 8.2. Developing Effective Sham Procedures 

Developing effective sham procedures for tFUS studies involving psychiatric patients remains a significant challenge. Among the reviewed studies, all but one had a control group, and three of the six possible sham conditions, as described in Section 5, were used: positioning the transducer over the participant’s head without delivering the ultrasound stimulation [67,71], using a high-impedance cap [72], and defocusing the acoustic wave [44,45]. More importantly, only two studies [72] report at least partial quantitative evaluations of their participants’ level of blinding: in one case [72], however, 100% of the participants receiving the active stimulation believed that they were receiving it, while 43.75% of the participants in the sham arm believed they were receiving active tFUS. In the other study [65], in the subsample of participants (N = 13/22) investigated, the sham and active sessions were not distinguishable. Variability in sham procedures and a lack of blinding assessments complicated the distinction between tFUS and placebo responses, especially in psychiatric populations [28]. 

Furthermore, an ideal and reproducible sham condition is yet to be developed for tFUS studies. Some features related to the procedure, such as the auditory perception of pulsed ultrasound or scalp overheating, can affect not only the blinding, but also the understanding of tFUS-specific neural effects [74,75] in studies involving both clinical and non-clinical populations. Future tFUS studies should therefore include a statistical evaluation of the degree of blinding of the participants to the condition and clarify which neurobiological responses are tFUS-specific and which are related to sensory confounds. This will hopefully lead to the implementation of optimal sham procedures for tFUS studies that are reliable and reproducible. 

### 8.3. Performing Large-Sample, Neurobiologically Informed tFUS Studies in Psychiatric Populations 

In psychiatric patients, the findings from most of the tFUS studies published so far, which include case reports and small, open-label trials, need to be considered preliminary, given that the modest sample sizes of these studies limit their clinical interpretability. Future studies in larger cohorts of individuals with psychiatric disorders are needed to confirm the improvements in the clinical symptoms reported in this review and to assess their stability over time. It would also be critical to collect neuroimaging and neurophysiological measures to help reveal the neural mechanisms underlying the effects of tFUS on clinical symptomatology in psychiatric patients. Specifically, combining tFUS with fMRI and EEG would allow for real-time monitoring and/or acute assessment of the stimulation effects, thus offering insights into the neural mechanisms underlying its therapeutic benefits in psychiatric populations. 

Future research should also focus on optimizing tFUS protocols for clinical use, especially by determining the most effective stimulation parameters to achieve appropriate inhibitory vs. excitatory effects. This could involve performing dose–response studies and identifying personalized computational modeling and/or stimulation approaches tailored to each patient’s anatomical and physiological characteristics.

## 9. Conclusions

In conclusion, tFUS represents a safe, novel, and promising approach in neuromodulation, particularly in neuropsychiatric disorders, in which deep brain regions play a critical role. While preliminary studies have provided encouraging results, the field is still in its infancy, with much work needed to standardize protocols and demonstrate consistent efficacy. Future advancements in real-time monitoring, individualized dosing, and sham control designs will likely shape the trajectory of tFUS as a viable clinical tool. We acknowledge that, at this stage, tFUS is comparatively difficult to implement and is expensive. However, it is currently the only non-invasive neuromodulation option available to reach deep brain structures. We also hope that it will become easier to use and more affordable in the near future. With ongoing research and collaborative efforts to address these challenges, tFUS has the potential to transform psychiatric care, offering a non-invasive, precise, and effective means of targeting both superficial and deep brain regions, thus potentially bridging a crucial gap in the current non-invasive brain stimulation landscape.
